# Assessing heart rate variability in type 1 diabetes mellitus—Psychosocial stress a possible confounder

**DOI:** 10.1111/anec.12760

**Published:** 2020-04-30

**Authors:** Eva Kristiansen, Pär Wanby, Karin Åkesson, Peter Blomstrand, Lars Brudin, Johanna Thegerström

**Affiliations:** ^1^ Department of Pediatrics Region Kalmar County Kalmar Sweden; ^2^ Department of Medicine and Optometry Linnaeus University Kalmar Sweden; ^3^ Department of Internal Medicine Section of Endocrinology Region Kalmar County Kalmar Sweden; ^4^ Futurum – Academy for Health and Care Jönköping Sweden; ^5^ Department of Pediatrics Region Jönköping County Jönköping Sweden; ^6^ Department of Natural Science and Biomedicine School of Health and Welfare Jönköping University Jönköping Sweden; ^7^ Department of Clinical Physiology Region Jönköping County Jönköping Sweden; ^8^ Department of Clinical Physiology Region Kalmar County Kalmar Sweden; ^9^ Present address: Department of Pediatrics The Queen Silvia Children's Hospital Sahlgrenska University Hospital Region Västra Götaland County Gothenburg Sweden

**Keywords:** cortisol, diabetic autonomic neuropathy, heart rate variability, Holter monitoring, psychosocial stress, type 1 diabetes mellitus

## Abstract

**Background:**

Autonomic neuropathy (AN) commonly arises as a long‐term complication in diabetes mellitus and can be diagnosed from heart rate variability (HRV), calculated from electrocardiogram recordings. Psychosocial stress also affects HRV and could be one of several confounders for cardiac AN. The present work investigated the impact of psychosocial stress on HRV in individuals with type 1 diabetes mellitus (T1DM) and assessed the use of salivary cortisol as a biomarker for psychosocial stress in this context.

**Methods:**

A total of 167 individuals 6–60 years old (113 with T1DM and 54 healthy controls) underwent 24‐hr ECG recordings with HRV analysis. Salivary cortisol was sampled thrice during the registration day. Perceived psychosocial stress along with other factors of possible importance for the interpretation of HRV was documented in a diary.

**Results:**

Heart rate variability (high‐frequency power during sleep) was reduced (*p* < .05) with older age, longer diabetes duration, higher mean glucose levels, physical inactivity, and perceived psychosocial stress. Salivary cortisol levels in the evening were increased (*p* < .05) in women in ovulation phase, in individuals with preceding hypoglycemia or with hyperglycemia. The amplitude of salivary cortisol was reduced (*p* < .05) with the presence of perceived psychosocial stress, but only in adult healthy controls, not in individuals with diabetes.

**Conclusion:**

Psychosocial stress might be a confounder for reduced HRV when diagnosing cardiac AN in T1DM. Salivary cortisol is, however, not a useful biomarker for psychosocial stress in diabetes since the physiological stress of both hypoglycemia and hyperglycemia seems to overrule the effect of psychosocial stress on cortisol.

## 1 INTRODUCTION

Reduced heart rate variability (HRV) as a sign of cardiac autonomic dysfunction has been found to be an independent predictor of death and of cardiovascular events, in individuals with diabetes mellitus, and in individuals with different heart conditions (Ewing, Campbell, & Clarke, 1976; Maser, Mitchell, Vinik, & Freeman, 2003; Vinik, Casellini, Parson, Colberg, & Nevoret, 2018). Conversely, high HRV has been linked to longevity in a healthy population (Zulfiqar, Jurivich, Gao, & Singer, 2010).

Damage to the small nerve fibers of the autonomic nervous system, autonomic neuropathy, occurs in diabetes mellitus as a long‐term effect of hyperglycemia (Kamenov & Traykov, 2012). The long fibers of the vagus nerve, including the parasympathetic fibers supplying the heart, might be particularly sensitive to damage and therefore affected early on (Breiner, Lovblom, Perkins, & Bril, 2014), impacting HRV.

Traditionally, a combination of autonomic reflex tests has been used to diagnose cardiac autonomic neuropathy (CAN) (Ewing, 1981). In 1984, Ewing et al presented a study which suggested that assessment of cardiac parasympathetic indices using 24‐hr electrocardiographic (ECG) recordings might be more sensitive than the described reflex tests in detecting early cardiac parasympathetic damage in individuals with diabetes mellitus (Ewing, Neilson, & Travis, 1984). Since then, several studies have demonstrated a reduced HRV in individuals with type 1 diabetes mellitus (T1DM) compared to healthy individuals, with longer diabetes duration and poor glycemic control as prominent risk factors (Chessa et al., 2002; Jaiswal et al., 2013). Reduced HRV has been reported in children and adolescents with T1DM with very short duration (<1–2 years) in a few older studies (Pfeifer et al., 1984; Wawryk, Bates, & Couper, 1997). The more recent SEARCH for Diabetes in Youth Cohort Study found an incidence of CAN in young adults (18 years old) with T1DM of 12%, based on HRV from a 5‐min ECG recording (Jaiswal et al., 2017).

The analysis and the interpretation of HRV parameters, whether from short‐ or long‐term ECG recordings, pose some challenges. Although recommendations for standards of measurements for HRV were published in 1996 (“Heart rate variability: standards of measurement, physiological interpretation, and clinical use. Task Force of the European Society of Cardiology and the North American Society of Pacing and Electrophysiology,” 1996), there is no consensus regarding age‐specific normal ranges of HRV indices from long‐term ECG, which is a disadvantage when studying young individuals, and essential in developing a method for screening purposes and diagnosing CAN on an individual level.

Another methodological difficulty is the presence of possible confounders for reduced HRV, the most important of which is that of psychosocial stress. In the research fields of psychiatric conditions such as clinical depression, or that of biological psychology and stress research, HRV has emerged as an important tool to measure the physiological response to stress, in adults and in children (Kim, Cheon, Bai, Lee, & Koo, 2018). Several studies show that clinical depression, but also general stressors in life, reduces HRV in both adults and children (Knorr, Vinberg, Kessing, & Wetterslev, 2010; Michels et al., 2013).

Children and young individuals with diabetes are exposed to additional stressors in life compared with healthy individuals, in part due to the practical everyday management of the disease, but also due to an additional psychological burden simply because of living with a chronic disease (Chao et al., 2016; Hema et al., 2009). Therefore, when assessing the possible presence of early CAN with 24‐hr ECG and HRV analysis, accounting for psychosocial stress seems to be of importance in order to avoid false‐positive results. No previous studies on HRV in young persons with diabetes have taken psychosocial stress into consideration.

Salivary cortisol measurements are frequently used in scientific studies as a biological marker for psychosocial stress both in adults and in children (Bosch et al., 2009; Michels et al., 2013). Cortisol is the product of the hypothalamic–pituitary–adrenal axis (HPA) which responds to both physiological stress and psychosocial stress (Adam et al., 2017). Cortisol levels normally follow a circadian rhythm with a peak at awakening and declining levels during the day. The salivary sampling method is attractive compared with blood sampling, especially in children, since it is non‐invasive and pain free, and reliably mirrors cortisol blood levels (Aardal & Holm, 1995; Michels et al., 2013). Evening or midnight cortisol, cortisol awakening response (CAR), and the diurnal cortisol slope are commonly used measurements (Wang et al., 2014).

The HPA axis has been studied in subjects with diabetes with varying results (Sharma, Wigham, & Veldhuis, 2014), but salivary cortisol has only been used in a limited number of studies on individuals with T1DM in relation to stress. High midnight salivary cortisol was linked to depression, smoking, physical inactivity, and season in adults with T1DM in one meta‐analysis (Melin, Thunander, Landin‐Olsson, Hillman, & Thulesius, 2014), and flattened cortisol slope was associated with stress and subjective socioeconomic disadvantage in older adolescents with T1DM in another study (Zilioli, Ellis, Carre, & Slatcher, 2017).

Previously described factors of possible importance for the HRV analysis other than psychosocial stress are as follows: hypoglycemia (Jaiswal et al., 2014; Koivikko et al., 2012), physical activity (Shin et al., 2014), thyroid disease (Kilic, Gulgun, Tascilar, Sari, & Yokusoglu, 2012) and ovulation in fertile women (Weissman et al., 2009).

The present cross‐sectional methodological work, which is part of a larger prospective study, examines several individual and situational factors during a 24‐hr ECG registration that might need to be taken into account when interpreting HRV. It focuses on elucidating the usefulness of salivary cortisol as a biomarker of psychosocial stress in the context of assessing reduced HRV in individuals with T1DM.

## 2 MATERIAL AND METHODS

### 2.1 Subjects

Children and adolescents 6–18 years old with T1DM from the pediatric departments at three different hospitals in the southeast region of Sweden (Jönköping Ryhov County Hospital, Kalmar County Hospital, and Västervik Hospital) were enrolled consecutively in the study between May 2015 and June 2017 (*n* = 68). Approximately 18% of the children attending the diabetes clinics accepted to participate. Adults 19–60 years old with T1DM were enrolled consecutively from the department of internal medicine, diabetes, and endocrinology, Kalmar County Hospital, between May 2016 and February 2019 (*n* = 45, including 8 cases of late autoimmune diabetes in adults, LADA). Retrieving data from the Swedish National Diabetes Register (NDR), a comparison of HbA1c showed that study participants in the child and adolescent group had a lower mean HbA1c than non‐participants (56.4 versus 60.6, *p* < .05); that is, the study cohort mainly consisted of individuals with good or acceptable metabolic control. HbA1c did not differ significantly between adult participants and non‐participants (64.9 versus 67.1, *p* = .3). Healthy controls consisted of friends or partners of the patients (siblings were not allowed to participate in accordance with the ethics committee's directions). Additional 10–18‐year‐old healthy controls were recruited from schools in Kalmar, by school nurses in spring 2019. Exclusion criteria were known heart disease, neurological disease, systemic disease or treatment with systemic steroids for any reason, impaired kidney function, depression, or medically treated sleeping disorders, assessed by the treating physician in inclusion form (subjects with T1DM), or self‐reported (denied) in inclusion form (controls). Subjects with well‐controlled asthma or allergy (*n* = 8) and subjects with stable thyroid substitution (*n* = 9) were included. Body mass index (BMI) was calculated from weight and height measured at the nearest clinical visit or was self‐reported. Adult participants were classified as overweight if BMI ≥25. For children, ISO‐BMI 25 was used, according to the Swedish pediatric society for endocrinology and diabetes (Karlberg, Luo, & Albertsson‐Wikland, 2001).

### 2.2 ECG recordings and HRV analysis

Twenty‐four‐hour ECG registrations were performed with the Cardiospy device EC‐3H 3‐channel Holter ECG system (Labtech Ltd). ECG recordings started between 3 and 6 p.m. and were of 22‐ to 24‐hr duration. Sampling rate was 256 Hz. ECG analysis was made using the Labtech Ltd Cardiospy software version 5.04.01. Algorithm‐based automatic identification of QRS complexes and identification of disturbances and ectopic beats were followed by a manual analysis and strict removal of all interferences by an experienced biomedical scientist to leave only normal RR intervals for the HRV analysis. Analysis of both time domain (standard deviation of NN intervals [SDNN], root mean square of successive RR interval differences [rMSSD], and percentage of successive RR intervals that differ by more than 50 ms [pNN50])and frequency domain (power of the low‐frequency band [LF] and the high‐frequency band [HF], 0.04–0.15 and 0.15–0.4 Hz, respectively (Shaffer & Ginsberg, 2017)) were made (Table 3). All HRV parameters were found to be highly interdependent, and for further correlation analysis, the natural logarithm of the high‐frequency power spectral density component during sleep, Ln(HFPower sleep), was chosen to represent HRV. The study did not aim to diagnose CAN on an individual level, but compared HRV between groups.

### 2.3 Diary

The participants were provided with a standardized but open form of diary, and instructed to enter time and nature of different activities during the registration, in particular physical activities and all forms of stress or other events of emotional impact.

Self‐reported stress entries in the diary of short duration (<5 min, e.g., “hurrying to catch the bus”) were disregarded. Stress comprised occupational, school, or various performance stress, as well as expressed emotions such as anger, irritation, sadness, worry, or anxiety. The presence of psychosocial stress was used as a dichotomous variable, but duration of stress was also estimated in minutes in order to roughly compare stress burden between groups. Emotions associated with hypoglycemia were not classified into the psychosocial stress category, but hypoglycemia was regarded as a separate entity of physiological stress.

Because the recording device contained an accelerometer, visualization, relative quantification, and exact time recording of physical activity were possible. In this study, being physically active was defined as recorded movement and/or a diary entry by the patient describing physical activity, along with an increase in heart rate of >40% from resting heart rate, and a HR >100 bpm. The sum of physically active time (min) was recorded for each participant. A cutoff at 100 min/registration day was used for the dichotomous variable “physical activity.”

Exact time for going to bed and for awakening was obligatory, as well as an estimation of the subjective quality of sleep. “Impaired sleep” (dichotomous variable) was used as a stress category in the analysis.

The participants were additionally instructed to write down the amount of ingested beverages containing caffeine (coffee, tea, coke, or other energizers). The amounts were converted to caffeine equivalent doses. Participants were also asked to note medication taken within 48 hr before start and during the registration day.

Women and girls after the onset of menarche were asked to estimate whether they were within two days of ovulation and to note the date of the first day of their last menstruation.

### 2.4 Cortisol sampling

Cortisol was collected with the oral swab method (SalivaBio^®^, Salimetrics^®^ LLC). Patients were instructed to abstain from eating, drinking, and toothbrushing one hour before sampling. Samples were kept in a refrigerator until returned to the laboratory where the swabs were centrifuged at 700 g for 10 min to recover saliva. Samples were kept frozen at −20°C until analyzed by competitive enzyme‐linked immunoassay from Salimetrics^®^ LLC, at the Department of Clinical Chemistry, Linköping University (Aardal & Holm, 1995). Each participant collected a series of three salivary samples at home: an evening sample, collected within 1 hr before going to bed; a morning sample, collected directly at awakening; and a second morning sample, collected 30 min after the first morning sample in order to calculate the cortisol awakening response (CAR). Cortisol amplitude was calculated as the difference between the highest value of the two morning samples and the evening cortisol. We preferred the term “cortisol amplitude” to “diurnal cortisol slope” which is often used, although under various definitions (Adam et al., 2017), because the evening salivary sample preceded the morning samples in our study. Especially the morning samples are known to be sensitive to correct timing of the sampling (Stalder et al., 2016). Adequate collection timing in this context was assured by (a) a precise entry of time in the diary, (b) pressing the event button on the recording device at the time of collecting the salivary samples, and (c) comparing given times with movement recordings on the ECG registration. Only if there was a congruency between either exact time entries in the diary or event entries in the ECG with the movement pattern and increased heart rate (indicating awakening) were the morning samples included in the analysis. Based on this strict selection, 83% of the patients had acceptable cortisol samples and were included in the analysis (167 out of 201 individuals). Individuals with diabetes had a lower rate of successful sampling than controls (80% versus 88%), mostly due to low glucose levels in the morning that impeded cortisol sampling in some cases. Children had a lower rate of successful sampling than adults (80% versus 91%).

### 2.5 Glucose monitoring

If a participant used a subcutaneous continuous glucose monitoring device (CGM) as a tool for their everyday management of diabetes, and they agreed to share the data, these data were used. Otherwise, participants were asked to supply all manually acquired glucose values from the memory of their testing device or any other form of glucose recorder, or diary, 7 days back before the start of the ECG registration. Half of the group consisting of children and adolescents had CGM data, whereas only approximately one fourth of the adults had CGM.

Hypoglycemia was defined as a glucose below 3.5 mmol/L if associated with typical symptoms noted in the diary. If asymptomatic, a cutoff at 3 mmol/L was used, and on CGM, two measurements at least 15 min apart were required. These cutoffs are slightly stricter compared to a clinical setting where a glucose below 3.9 mmol/L is cause to take action (Ly et al., 2014), but were based on levels when hypoglycemia might influence HRV (Koivikko et al., 2005).

Mean glucose (mmol/L) preceding the registration was calculated seven days back. CGM rendered between 168 and 2 016 entries/7 days, depending on sampling rate. Mean number of manual entries/7 days was 45 (range 8–121).

HbA1c values (mmol/mol, International Federation for Clinical Chemistry and Laboratory Medicine, IFCC) were derived from the patients’ records. The measurement nearest in time to the registration, taken during a routine clinical visit, was chosen.

### 2.6 Statistics

**FIGURE 1:**
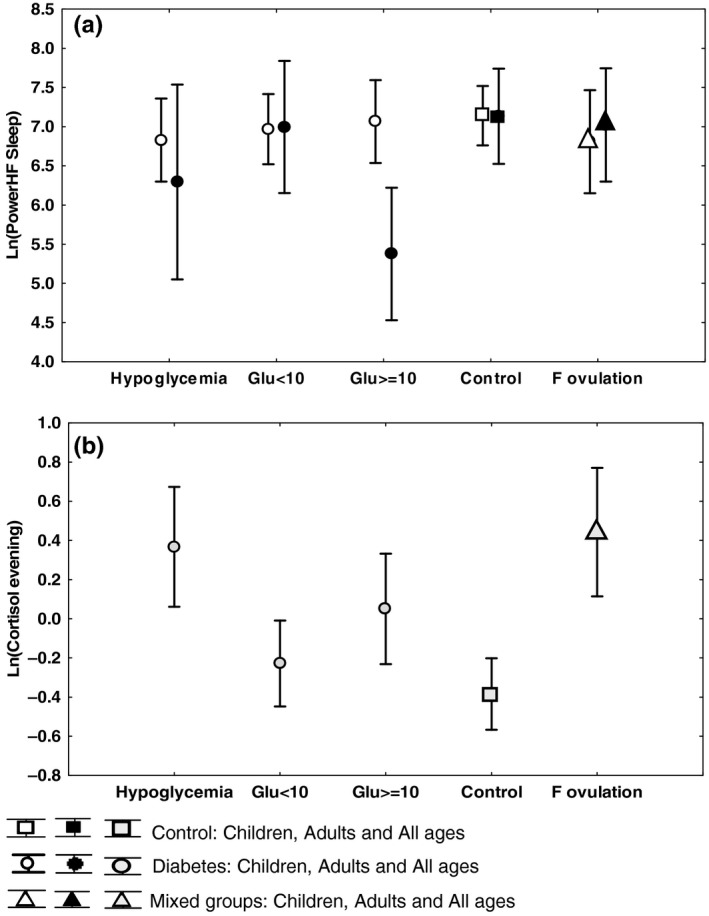
Metabolic and hormonal factors influencing heart rate variability (HRV) and cortisol Means and 95% confidence intervals shown. (a) HRV (LnHFPower Sleep), children, and adults (b) Cortisol (LnCortisol evening), whole group (since patterns for children and adults were the same). Horizontal axis from left to right: diabetes with hypoglycemia, diabetes with mean glucose <10 mmol/L, diabetes with mean glucose ≥10 mmol/L, controls and females with ovulation. Observations: Adults with a mean glucose ≥10 mmol/L had significantly reduced HRV compared with all other groups (*p* = .012), except adults with hypoglycemia (upper panel). Females in ovulation phase, individuals experiencing hypoglycemia, and diabetics with a mean glucose ≥10 mmol/L all had a higher cortisol in the evening than controls (*p* < .05, lower panel)

Mean values (*SD*) and medians (range) were used to describe the continuous variables for the four groups (consisting of diabetes and controls, subdivided into children and adults). Frequencies and percentages were used to describe the categorical variables. The Mann–Whitney *U* test or the Fisher exact test was used for comparisons between groups in Tables 1, 2, 3. *p* < .05 was considered statistically significant, but *p*‐values in Tables 1 and 2 are only shown for descriptive and not inferential purposes. Continuous variables were transformed to natural logarithms when appropriate (high‐frequency [HF] and low‐frequency [LF] power and cortisol parameters), allowing analyses of variance (ANOVAs) and analysis of covariance (ANCOVA) to be used. When HRV constituted the dependent variable, all statistical tests were corrected for heart rate (HR) (Kazmi et al., 2016) and/or age when appropriate. When cortisol constituted the dependent variable, all statistical tests were corrected for sex and age. Individuals experiencing hypoglycemia preceding a cortisol sample and women in ovulation phase (including all fertile women who were not able to provide a reliable date for their last menstruation and therefore to be safely said to be non‐ovulating) were excluded from other analysis of cortisol (see Sections 2.2.1, 2.2.3, and Figure 1). Non‐parametric tests (Wilcoxon test) were used for a sub‐analysis in Section 2.1.3. 

All statistical analysis was made using the program Dell Statistica, version 13 (Dell Inc.).

**TABLE 1:** Subject details at inclusion

Variable	Children/Adolescents			Adults		
	Controls	Diabetes	Diff (*p*)	Controls	Diabetes	Diff (*p*)
*N*	36	68		18	45	
Age (years)						
Mean (*SD*)	14.0 (2.4)	13.7 (2.8)		36.7 (11.1)	44.3 (12.1)	
Median (range)	14 (7–19)	14 (7–18)	.836	35 (23–59)	47 (20–60)	.036
Sex						
F (%)	21 (58.3)	33 (48.5)		14 (77.8)	29 (64.4)	
M (%)	15 (41.7)	35 (51.5)	.411	4 (22.2)	16 (35.6)	.379
Height (cm)						
Mean (*SD*)	164.2 (9.4)	161.6 (16.0)		168.4 (9.3)	170.3 (7.4)	
Median (range)	166 (140–179)	164 (123–198)	.618	168 (151–189)	170 (154–183)	.334
Weight (kg)						
Mean (*SD*)	56.4 (12.3)	55.9 (16.0)		73.5 (16.6)	75.4 (11.3)	
Median (range)	56 (32–78)	57 (25–86)	.929	69 (51–122)	75 (53–107)	.288
BMI (kg/m^2^)						
Mean (*SD*)	20.2 (2.9)	20.9 (3.7)		25.8 (4.0)	26.0 (3.3)	
Median (range)	19 (16–25)	21 (13–32)	.575	25 (20–34)	26 (20–36)	.744
Age at debut						
Mean (*SD*)	—	8.1 (3.7)		—	15.7 (10.7)	
Median (range)	—	8.0 (1.3–17.3)	—	—	13.0 (1.9–42.3)	—
Duration (years)						
Mean (*SD*)	—	5.6 (3.5)		—	28.6 (11.8)	
Median (range)	—	5.4 (0.4–15.0)	—	—	28.0 (10.0–52.4)	—
HbA1c (mmol/mol)						
Mean (*SD*)	—	55.0 (10.1)		—	58.1 (14.2)	
Median (range)	—	54 (37–92)	—	—	58 (28–106)	—
Glucose mean 7 days (mmol/L)						
Mean (*SD*)	—	9.0 (1.7)		—	9.9 (3.1)	
Median (range)	—	8.9 (5.3–12.9)	—	—	9.4 (5.3–18.4)	—

Abbreviations: BMI, body mass index; *SD*, standard deviation.

**TABLE 2:** Subject details from diary and registration on registration day

Variable	Children/Adolescents			Adults		
	Controls	Diabetes	Diff (*p*)	Controls	Diabetes	Diff (*p*)
*N*	36	68		18	45	
Physical activity						
Low (%)	22 (61.1)	43 (63.2)		14 (77.8)	42 (93.3)	
High (%)	14 (38.9)	25 (36.8)	.835	4 (22.2)	3 (6.7)	.095
Psychosocial stress						
No/Short (%)	24 (66.7)	39 (57.4)		7 (38.9)	24 (53.3)	
Stress (%)	6 (16.7)	21 (30.9)	.389	10 (55.6)	18 (40.0)	.222
Impaired sleep^a^	8 (22.2)	11 (16.2)	.440	6 (33.3)	12 (26.7)	.758
Stress (min)						
Mean (*SD*)	9.9 (11.1)	12.2 (15.2)		27.4 (31.8)	23.2 (30.3)	
Median (range)	5 (5–50)	5 (5–90)	.444	19 (5–110)	15 (5–185)	.757
Length of sleep (hr)						
Mean (*SD*)	8.0 (1.0)	8.3 (1.4)		7.3 (1.3)	7.3 (1.0)	
Median (range)	8.1 (4.8–9.7)	8.3 (5.5–13.8)	.371	6.9 (6.0–10.3)	7.2 (5.5–9.8)	.551
Hypoglycemia						
No (%)	—	40 (58.8)		—	34 (75.6)	
Yes (%)	—	28 (41.2)		—	11 (24.4)	

Abbreviations: *SD*, standard deviation.

^a^ Note that “Impaired sleep” partially overlaps with psychosocial stress.

**TABLE 3:** Results from ambulatory ECG and salivary cortisol measurements

Variable	Children/Adolescents			Adults		
	Controls	Diabetes	Diff (*p*)	Controls	Diabetes	Diff (*p*)
*N*	36	68		18	45	
Results ambulatory ECG						
HR						
Mean (*SD*)	81.1 (7.2)	84.2 (8.7)		75.9 (5.5)	79.3 (9.4)	
Median (range)	81 (67–96)	85 (64–104)	0.045	75 (64–86)	78 (58–99)	0.279
HR night						
Mean (*SD*)	65.0 (6.9)	67.6 (8.4)		61.2 (4.8)	66.5 (9.5)	
Median (range)	65 (50–76)	67 (49–92)	0.171	61 (52–71)	67 (47–87)	0.019
SDNN 24 hr						
Mean (*SD*)	175.7 (40.3)	166.9 (36.3)		166.3 (36.3)	135.8 (40.9)	
Median (range)	172 (110–291)	169 (87–249)	0.385	164 (117–252)	126 (56–232)	0.005
rMSSD sleep						
Mean (*SD*)	84.8 (37.2)	78.8 (38.4)		59.8 (32.7)	32.7 (19.9)	
Median (range)	80 (29–163)	70 (24–199)	0.412	47 (18–145)	29 (4–85)	0.001
pNN50 sleep						
Mean (*SD*)	41.6 (17.0)	37.2 (18.7)		28.9 (18.1)	12.1 (12.8)	
Median (range)	42 (7–75)	37 (4–74)	0.286	27 (1–67)	8 (0–46)	<0.001
Ln(PowerLF Sleep)						
Mean (*SD*)	7.5 (0.6)	7.3 (0.7)		7.2 (0.7)	6.2 (1.3)	
Median (range)	7.6 (6.3–8.7)	7.4 (5.8–8.8)	0.221	7.1 (5.8–8.3)	6.7 (2.6–8.6)	0.007
Ln(PowerHF Sleep)						
Mean (*SD*)	7.4 (0.8)	7.2 (0.9)		6.5 (1.0)	5.4 (1.5)	
Median (range)	7.2 (6.1–8.9)	7.4 (5.3–9.2)	0.424	6.4 (4.9–8.5)	5.5 (1.6–7.5)	0.004
Result cortisol						
Cortisol evening (nmol/L)						
Mean (*SD*)	1.0 (1.1)	1.2 (1.1)	*	1.0 (0.5)	1.4 (1.1)	*
Median (range)	0.7 (0.2–6.8)	1.0 (0.2–7.4)	0.067	1.0 (0.3–2.1)	1.2 (0.3–5.8)	0.113
Cortisol morning (nmol/L)						
Mean (*SD*)	7.9 (3.1)	8.6 (3.7)	*	10.0 (5.0)	8.2 (4.2)	*
Median (range)	7.7 (1.5–16.5)	7.9 (1.7–21.0)	0.561	8.9 (2.6–20.9)	7.4 (2.2–22.1)	0.176
Cortisol 30 min (nmol/L)						
Mean (*SD*)	12.0 (4.2)	12.8 (6.0)	*	15.9 (4.7)	12.0 (4.8)	*
Median (range)	11.0 (6.5–27.0)	12.4 (3.0–33.0)	0.570	14.2 (8.9–23.2)	11.7 (3.9–25.3)	0.006

Abbreviations: ECG, electrocardiogram; HR, heart rate; pNN50, percentage of successive RR intervals that differ by more than 50 ms; rMSSD, root mean square of successive RR interval differences; *SD*, standard deviation; SDNN, standard deviation of NN intervals.

* *p*‐values should be interpreted with caution for cortisol parameters since subjects with hypoglycemia and women with possible ovulation are included in these raw data (see Sections 1.6, 2.2.1, and 2.2.3).

## 3 RESULTS

No differences in baseline characteristics were found between participants with diabetes and controls in the children/adolescent group, whereas adult participants with diabetes were slightly older than controls with a tendency of being more physically inactive (Tables 1 and 2). Raw data for HRV and cortisol are shown in Table 3.

### 3.1 Heart rate variability

Adults with diabetes had significantly lower time domain and frequency domain HRV parameters than controls (Table 3). HRV represented by the natural logarithm of the high‐frequency band (HF) during sleep, Ln(PowerHF Sleep), was significantly reduced in the diabetes group also when corrected for age, sex, and physical activity (*p* = .022). There was no significant difference in HRV between groups in children (*p* = .424).

#### 3.1.1 HRV and constitutional factors

Heart rate variability correlated with resting heart rate (HR) at the corresponding night (*p* < .001). HR, in turn, correlated with anthropomorphic data in children (length). Overweight did not correlate significantly with HRV when corrected for age, since the prevalence of overweight was more than twice as high in 35‐ to 60‐year‐old subjects (57%) compared with other age groups. There was no difference between sexes after correction for HR and age. Menstrual cycle as in ovulation did not affect HRV (as opposed to cortisol, see 2.2.1 and Figure 1). HRV decreased with age, both in healthy controls and in individuals with diabetes (*p* < .001).

#### 3.1.2 HRV and metabolic factors

The duration of disease correlated with decreasing HRV in persons with diabetes, regardless of the effect of aging (*p* = .033). Individuals with a high mean glucose level at short term (≥10 mmol/L) tended to have lower HRV parameters than those with lower mean glucose levels, but this was only significant in adults (*p* = .012; Figure 1). Children 12 years or younger (*n* = 25) showed no correlation at all between HRV and HbA1c levels. Adolescents on the one hand and adults on the other hand differed significantly (*p* = .016) in their respective relationships between HRV and HbA1c, adults with HbA1c >55 mmol/mol displaying lower HRV than those with HbA1c <55mmol/mol, and adolescents showing an opposite trend.

#### 3.1.3 HRV and possible confounders

Hypoglycemia did not significantly impair whole night HRV on a group level (Figure 1). Evaluating individuals with hypoglycemia during the night (*n* = 12) and comparing the lowest hourly measurement of HRV during hypoglycemia with whole night mean HRV, the variability was shown to be significantly reduced during hypoglycemia (*p* = .002, Wilcoxon test). The heart rate during the hour of hypoglycemia, however, was significantly higher compared with the mean heart rate of the night (*p* = .002, Wilcoxon test).

**FIGURE 2:**
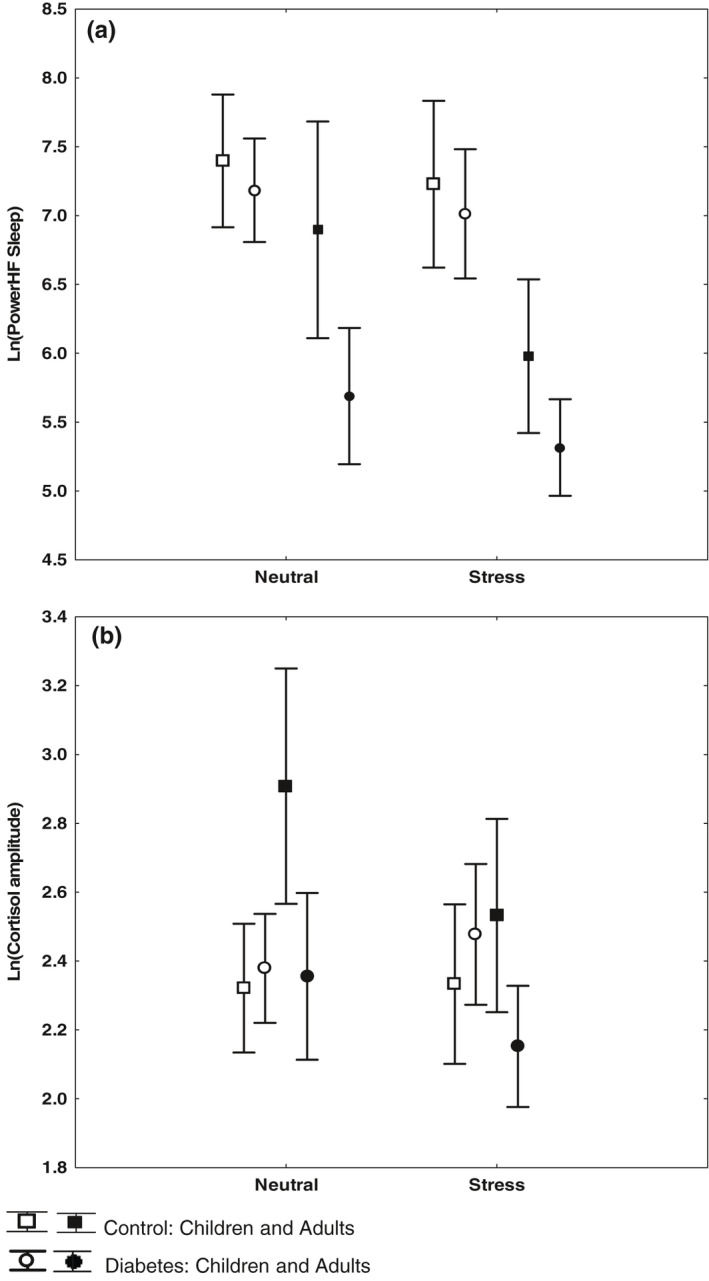
Effect of psychosocial stress on HRV and cortisol Means and 95% confidence intervals shown. Comparison of (a) HRV (LnHFPower Sleep) and (b) cortisol (LnCortisol evening) between two groups: “Neutral”: individuals who reported no stress in their diary and who had acceptable subjective sleep quality and “Stress”: individuals who reported some sort of psychosocial stress and/or reported impaired sleep quality. Ovulating women and individuals experiencing a hypoglycemia were excluded from analysis (see Figure 1). Effects are displayed for children and adults, diabetes, and controls separately. Observations: Total effect of psychosocial stress on HRV was significant (*p* < .05; upper panel), but only healthy adults differed significantly in cortisol amplitude between individuals experiencing stress and those who did not (*p* = .013, lower panel)

Psychosocial stress significantly reduced HRV (*p* < .05, Figure 2), in both healthy controls and in the diabetes group. Controls and individuals with diabetes experienced similar amounts of psychosocial stress, according to diary entries and episodes counted in minutes (Table 2). Children reported less stress than adults (*p* < .001), and women had a higher sum of stress minutes than men (*p* = .028). Subanalysis of different stress categories showed that the presence of occupational or school stress was most common and resulted in significantly lower HRV compared with stress neutral subjects (*p* = .011). Subjective experience of impaired sleep during the registration night, regardless of objective sleeping hours, also correlated significantly with lower HRV (*p* = .004), although not in healthy control children.

HRV was significantly higher in children/adolescents who were physically active more than 100 min/registration day than in those who were not (*p* = .010).

Being on thyroid substitution with stable hormone levels or being on maintenance therapy with SSRI, under the condition that the depression itself was in remission judged by the patient herself, did not affect HRV (studied only in a small subgroup of 36‐ to 60‐year‐old women). Minor complaints like taking pain killers for a headache or menstrual pain, medication for allergy or for well‐controlled asthma, or having a mild cold/minor infection did not seem to affect HRV (these factors were all grouped together and tested against subjects who reported none of these factors).

Caffeine intake did not influence whole night HRV. Season had no impact on either HRV or cortisol values.

### 3.2 Cortisol

Individuals with diabetes had significantly higher cortisol levels in the evening than controls (*p* = .010), but there was no significant difference between groups in CAR levels or in cortisol amplitude.

#### 3.2.1 Cortisol and gender

Up to the age of 12, there was no difference in cortisol levels between the sexes. Female adolescents and fertile women, however, had higher and more variable levels of cortisol than male adolescents and adult males (*p* = .010). Evening cortisol (Figure 1), morning cortisol 30 min after awakening, and the cortisol amplitude were all significantly elevated (*p* < .001 and *p* = .013) in females who might be ovulating around the time of the registration day (*n* = 16, 10 T1DM, six controls), as compared to both non‐ovulating females and males.

#### 3.2.2 Cortisol and sleep

Longer sleep (particularly in adolescents) lowered CAR (*p* = .002).

#### 3.2.3 Cortisol and glucose

A hypoglycemia preceding, but not too close to (>3 hr), the time point of sampling of saliva was found to give significantly higher cortisol levels as compared to euglycemic diabetic individuals (Figure 1). Thus, evening cortisol levels were affected by hypoglycemia the previous day (*p* = .001), but not in the same evening, and CAR was affected by early night hypoglycemias (*p* = .020), but not late‐night hypoglycemias.

Mean glucose levels above 10 mmol/L during the preceding 7 days to the registration gave higher basal cortisol levels than lower blood glucose levels (*p* = .044; Figure 1). Ovulating women with higher cortisol levels, however, had slightly lower glucose levels than non‐ovulating women (8.3 versus 9.8 mmol/L, *p* = .18, not significant).

#### 3.2.4 Cortisol and psychosocial stress

The cortisol amplitude was significantly lower in adult healthy control subjects who experienced some sort of stress during the registration day as compared to those who had made no note of stress in their diary (*p* = .013). None of the cortisol markers, however, correlated with stress in the diabetes group. The difference in cortisol amplitude between diabetics and controls was significant (*p* = .030; Figure 2).

## 4 DISCUSSION

To summarize the present work, HRV during sleep was found to decrease with older age, longer diabetes duration, higher mean glucose levels (in adults), physical inactivity (in children), and perceived psychosocial stress. Other situational factors like ovulation in women, BMI, caffeine intake, or hypoglycemia did not significantly affect HRV during sleep. Salivary cortisol levels in the evening were increased in women in ovulation phase, in individuals with preceding hypoglycemia, or with hyperglycemia. The amplitude of cortisol was reduced with the presence of perceived psychosocial stress, but only in adult healthy controls, not in individuals with diabetes.

These results can be useful when endeavoring to standardize a method for diagnosing CAN from HRV, avoiding confounders in the process.

A 24‐hr registration of ECG has the advantage over short‐term ECG containing extensive and complete data over a long time period. In analogy with 24‐hr blood pressure examinations, the long‐term ECG registrations are made in the patients’ homes or in their everyday environment. Especially when studying the autonomous nervous system, it is important to avoid the “white coat” effect which might bias the short‐term measurements of both adults and children in a hospital setting (Krmar, 2019; Pickering, Shimbo, & Haas, 2006). Examination conditions over all are less well controlled, however, in ambulatory examinations. Which exact conditions that must be controlled and what restrictions that are to be imposed on the patient during a 24‐hr ECG registration in order to obtain interpretable results of HRV is yet to be specified. The present study addressed some of these issues.

For a diagnostic method to be useful in everyday practice, especially if it were to be chosen as a screening tool, it is essential that it should be simple to apply and that it does not impose too many restrictions on the examined person's everyday life, nor limit too extensively the groups of patients possible to examine. This is the reason patients with stable thyroid substitution therapy were permitted to participate in the study. It is also why we permitted the intake of caffeine during the registration day and why the exclusion criteria for being on SSRI or beta‐stimulants were limited to patients with active disease, whereas maintenance therapy was allowed.

Thyroid disease is a common comorbidity of diabetes and has in itself been reported to lower HRV (Celik et al., 2011; Kilic et al., 2012). This renders it as a possible confounder when studying CAN. In relation to age and gender, stable thyroid substitution therapy did not seem to affect HRV significantly in our material. More data especially in the younger age groups are needed, however.

Psychiatric illness as in depressive and anxiety disorders is on the increase in Sweden (Hagquist, Due, Torsheim, & Valimaa, 2019). There are indications that clinical depression is overrepresented in individuals with diabetes as compared to a population without chronic disease (Andreoulakis, Hyphantis, Kandylis, & Iacovides, 2012; Wang et al., 2019). While there is evidence that active depression significantly lowers HRV (Paniccia, Paniccia, Thomas, Taha, & Reed, 2017), making it unsuitable to attempt to diagnose CAN at such a point of time in the patient's life, it is unclear whether this effect remains after remission of the disease. A substantial number of patients will remain on SSRI medication for several years after their actual depressive episode. Are they inept for assessment of HRV? Again, it was only possible to analyze a subgroup of patients, which however did not differ in HRV from their peers without SSRI medication.

A number of factors potentially interfering with the HRV analysis will mainly revert to a strong inverse correlation between HRV and HR (Billman, 2013). Small body size, being on medication such as beta‐stimulators for asthma or stimulants for neuropsychiatric disorders, caffeine intake, hypoglycemia with adrenal counter regulation, infection, pain, physical activity, and stress will all increase HR. Factors like nicotine and alcohol intake which also increase HR were not accounted for in this study. Studying nighttime HRV, which was done in the present work, with a resting HR, probably somewhat attenuates the impact of several of the above factors, yet some kind of correction for HR is highly recommended (Kazmi et al., 2016). It was relatively reassuring, however, in this study, to observe that caffeine intake did not affect whole night HRV parameters, nor did minor complaints of having a cold, transient pain, allergy problems, or well‐controlled asthma.

Hypoglycemia has been shown to lower HRV, at least in the short term (Koivikko et al., 2005, 2012). There are some indications that hypoglycemic stress over time in an individual might also reduce HRV in the long term (Jaiswal et al., 2014). In our material, even if hypoglycemia affected HRV (mainly by slightly elevated HR) in the short term (within the hour), it did not have a significant effect on whole night HRV. As a precaution, however, HRV should probably not be analyzed in patients experiencing long hypoglycemia before or during the registration. Physical activity did significantly impact HRV during the night in young individuals, which is in concordance with previously published data on the beneficial effect of physical activity on HRV (Shin et al., 2014). As a well‐documented general recommendation (Perk et al., 2012), but also in the context of interpreting HRV, and diagnosing CAN, patients should therefore be encouraged to avoid physical inactivity.

Self‐reported psychosocial stress, as well as subjective poor sleep quality, correlated significantly with reduced HRV, even after correction for heart rate. This is in line with previous research on stress and HRV (Kim et al., 2018), but has not been demonstrated in individuals with diabetes before. Psychosocial stress should therefore be considered a possible confounder when diagnosing CAN from HRV. To control for psychosocial stress by classifying or grading its effects is difficult even when using questionnaires or structural interviews, however. An objective biological marker of stress would therefore be an attractive alternative.

While salivary cortisol is an extensively documented method and frequently used in stress research, its pitfalls, especially concerning the cortisol awakening response (CAR), are also well known (Stalder et al., 2016). Compliance with the timing of sampling is difficult, leading to exclusion of patients from the analysis. Although we took great pains to minimize sampling errors (see Section 1.4), the CAR measurement still had greater variations and was less useful than the evening cortisol and cortisol amplitude measurements. As an example, long sleepers who rose late in the morning (mostly adolescents) had lower CAR than their peers who woke up earlier. This is most probably due to partial awakenings and snoozing prior to collecting the morning salivary samples; that is, the actual morning peak of cortisol occurred before the conscious awakening.

Because ovulation proved to have a pronounced effect on both morning and evening cortisol levels, studying fertile women will add a methodological difficulty to the interpretation of salivary cortisol, in having to account for each woman's stage of the menstrual cycle. Adolescent girls are particularly difficult to assess since they are known to have scarce and irregular ovulations around menarche. One study indicated that HRV, too, might be affected by ovulation (Weissman et al., 2009), but nothing supported this theory in the present study.

Another study suggested that midnight cortisol might have a seasonal variation that should be taken into account (Melin et al., 2014). However, we found no correlation in this study between cortisol and the month or season the sampling was made.

In concordance with previous studies (Wang et al., 2014), we found the amplitude of cortisol (comparable to the diurnal cortisol slope (Adam et al., 2017)) to correlate with self‐reported stress in the healthy adult population, but no such correlation could be seen in the diabetes group.

Evening cortisol was higher in individuals with diabetes than in healthy controls, but psychosocial stress did not seem to be a plausible explanation for this. The paramount influence on cortisol in this material, except for the hormonal effects of ovulation, proved instead to be metabolic factors. On the one hand, hypoglycemia preceding a salivary sample was shown to increase the cortisol level, most likely due to its physiological counter regulatory function. On the other hand, hyperglycemia also correlated with higher cortisol levels. It is unlikely that hyperglycemia was caused by primarily elevated cortisol levels, since the case of ovulating women with high cortisol levels showed an opposite relationship (see Section 2.2.3). The influence of hyperglycemia on cortisol might be due to its pro‐inflammatory effects (Aburawi et al., 2016; Alvarez‐Almazan, Filisola‐Villasenor, Aleman‐Gonzalez‐Duhart, Tamay‐Cach, & Mendieta‐Wejebe, 2020). This is in line with a meta‐analysis of diurnal cortisol slopes and mental and physical health outcomes which showed that the strongest correlations with cortisol levels were found for inflammation and immune system outcomes (Adam et al., 2017).

## 5 LIMITATIONS

Study participants in the children/adolescent group consisted of individuals with better than the average HbA1c levels (see Section 1.1). This might explain why we found no HRV reduction related to metabolic factors in this group compared with the control group. This is gratifying in itself since it probably translates in a low prevalence of CAN in this group, although in the research setting, it entails that it will take longer to detect early signs of CAN in this cohort. Children and adolescents also reported significantly less stress than adults which weakened the power in the correlation tests between HRV and psychosocial stress in this age group. The method of subjectively reporting stress in a diary has its limitations and might be less suitable for children than adults, even if aided by their parents to fill in the diary. The fact that all four groups (children and adults, T1DM, and controls) showed the same trend and contributed to the significant overall effect of psychosocial stress on HRV (see Figure 2) strengthens the connection, however.

## 6 CONCLUSION

Psychosocial stress seems to be a factor to account for in the context of screening for CAN in individuals with T1DM by assessing HRV from 24‐hr ECGs. Cortisol levels are, however, of limited use when assessing psychosocial stress in individuals with diabetes, mainly because of an overriding effect of the physiological metabolic stress of T1DM itself over psychosocial stress.

## CONFLICT OF INTEREST

The authors have no conflicts of interest to declare.

## AUTHOR CONTRIBUTIONS

J.T. conceived the study. E.K. and J.T. processed all data, made initial analysis of results and wrote article draft. Statistical analysis was done by J.T. and L.B. who together with P.W., K.Å., and P.B. participated in discussions and contributed to the design and the implementation of the research and to the analysis of the results. All authors reviewed and commented on the manuscript

## ETHICAL APPROVAL

The Heart Rate Variability in Diabetes Study was approved by the Regional Ethics Committee of Linköping, Sweden. The study complies with the Helsinki Declaration. All participants provided their written informed consent.
